# Using Galaxy-P to leverage RNA-Seq for the discovery of novel protein variations

**DOI:** 10.1186/1471-2164-15-703

**Published:** 2014-08-22

**Authors:** Gloria M Sheynkman, James E Johnson, Pratik D Jagtap, Michael R Shortreed, Getiria Onsongo, Brian L Frey, Timothy J Griffin, Lloyd M Smith

**Affiliations:** Chemistry Department, University of Wisconsin-Madison, 1101 University Ave., Madison, WI 53706 USA; Minnesota Supercomputing Institute, University of Minnesota, 117 Pleasant St SE, Minneapolis, MN 55455 USA; Department of Biochemistry, Molecular Biology and Biophysics, University of Minnesota, 6-155 Jackson Hall, 321 Church Street SE, Minneapolis, MN 55455 USA; Center for Mass Spectrometry and Proteomics, University of Minnesota, 43 Gortner Laboratory, 1479 Gortner Avenue, St. Paul, MN 55108 USA; Genome Center, University of Wisconsin-Madison, 111 University Ave, Madison, WI 53705 USA

## Abstract

**Background:**

Current practice in mass spectrometry (MS)-based proteomics is to identify peptides by comparison of experimental mass spectra with theoretical mass spectra derived from a reference protein database; however, this strategy necessarily fails to detect peptide and protein sequences that are absent from the database. We and others have recently shown that customized proteomic databases derived from RNA-Seq data can be employed for MS-searching to both improve MS analysis and identify novel peptides. While this general strategy constitutes a significant advance for the discovery of novel protein variations, it has not been readily transferable to other laboratories due to the need for many specialized software tools. To address this problem, we have implemented readily accessible, modifiable, and extensible workflows within Galaxy-P, short for Galaxy for Proteomics, a web-based bioinformatic extension of the Galaxy framework for the analysis of multi-omics (e.g. genomics, transcriptomics, proteomics) data.

**Results:**

We present three bioinformatic workflows that allow the user to upload raw RNA sequencing reads and convert the data into high-quality customized proteomic databases suitable for MS searching. We show the utility of these workflows on human and mouse samples, identifying 544 peptides containing single amino acid polymorphisms (SAPs) and 187 peptides corresponding to unannotated splice junction peptides, correlating protein and transcript expression levels, and providing the option to incorporate transcript abundance measures within the MS database search process (reduced databases, incorporation of transcript abundance for protein identification score calculations, etc.).

**Conclusions:**

Using RNA-Seq data to enhance MS analysis is a promising strategy to discover novel peptides specific to a sample and, more generally, to improve proteomics results. The main bottleneck for widespread adoption of this strategy has been the lack of easily used and modifiable computational tools. We provide a solution to this problem by introducing a set of workflows within the Galaxy-P framework that converts raw RNA-Seq data into customized proteomic databases.

**Electronic supplementary material:**

The online version of this article (doi:10.1186/1471-2164-15-703) contains supplementary material, which is available to authorized users.

## Background

Mass spectrometry-based proteomics is widely employed to characterize proteins in myriad organisms, ranging from E. coli to human. Fundamental to almost all proteomics analyses is the database search step, where experimental peptide mass spectra are matched with theoretical peptide mass spectra derived from a protein reference database [1]. This MS database searching strategy relies on the completeness and quality of the protein reference database, meaning that peptides and proteins are only identified if their correct sequence is present in the protein reference file. However, individual organisms often possess genetic variations that differ from the canonical sequences present in the database. These variations are often not represented in the reference database causing the corresponding peptides to be invisible to MS-based analyses.

In recent years, high-throughput RNA sequencing has been used to empirically determine the transcript sequences expressed in a given sample, strain, cell line, or tissue, and has become accessible to many researchers [2, 3]. Taking advantage of this powerful new capability, we and others have developed novel strategies to leverage RNA-Seq for the detection of sample-specific protein variations [4–11]. In this strategy parallel RNA-Seq and proteomics data are collected from the same or related samples. Novel sequences discovered from RNA-Seq data are translated into proteins and added to the MS search database, which can then be employed to detect the corresponding protein variations.

RNA-Seq derived databases tailored for a given sample can improve proteomics in two main ways. First, and most importantly, RNA-Seq can be used to reveal novel single nucleotide polymorphisms (SNPs), indels, alternative splice forms, and gene fusions at the transcript level that, when translated, yield protein sequences that are not in the reference protein database. These novel protein sequences are then appended to the reference database and employed for MS-searching, enabling the detection of novel peptides. Second, RNA-Seq can be used to estimate the abundance of transcripts and this information can be used to improve database searching, such as through reduction of protein database size or through use of transcript abundances in calculating protein identification scores. We describe here a database reduction procedure in which RNA-Seq is used to quantify transcript levels and all protein entries in the database that fall below a threshold expression level for the corresponding transcript are removed [10, 12, 13]. This can be useful for reducing database size, but has the possible disadvantage of excluding proteins whose protein abundance levels are high but have low transcript abundance.

The greatest bottleneck in harnessing RNA-Seq data for the discovery of protein variations is not data generation-- deep coverage RNA-Seq data is readily and inexpensively produced--but rather in creating accessible and flexible bioinformatic pipelines to process the data. Given that sequencing platforms and software tools are rapidly evolving, researchers need an environment where it is easy to quickly integrate new transcriptomic and proteomic tools and readily modify workflows to suit their system of study. There is a dire need for transparency and sharing of workflows so that other labs can build upon prior work. These problems are magnified when considering the troves of next generation sequencing (NGS) data that are currently underutilized in the field of proteomics. One tool, CustomProDB, describes an R package to streamline the process of RNA-Seq-based database creation; however, we believe there is still a need for flexible tools that can be easily modified and integrated into larger bioinformatic pipelines [14].

Here we address the bioinformatic bottleneck in RNA-Seq-based protein database construction by introducing flexible, extensible, and sharable workflows within usegalaxyp.org, the public version of Galaxy-P. Galaxy-P is an extension of the original web-based Galaxy framework [15–17], with a focus on proteomic and multi-omic data analysis applications. We present three workflows that can be used for RNA-Seq-derived proteomic database construction. These workflows are transparent, easily shared, and flexible, so researchers, especially those without expertise in computer science and bioinformatics, can quickly extend and evolve the workflows for their needs. We describe the workflows and show their utility in discovering novel peptides in both human (Jurkat cells) and mouse (pancreatic islet) samples. The implementation of these workflows in Galaxy-P will help researchers utilize NGS data for the detection and discovery of protein variations via mass spectrometry.

## Results and discussion

### Galaxy workflows

We have developed workflows in Galaxy-P that convert RNA-Seq data into three types of readily usable proteomic databases. These are databases containing novel single amino acid polymorphisms; databases containing novel splice junction sequences; and a reduced database, which only contains protein sequences with corresponding transcripts that are expressed over a threshold level of abundance.

**Figure 1:**
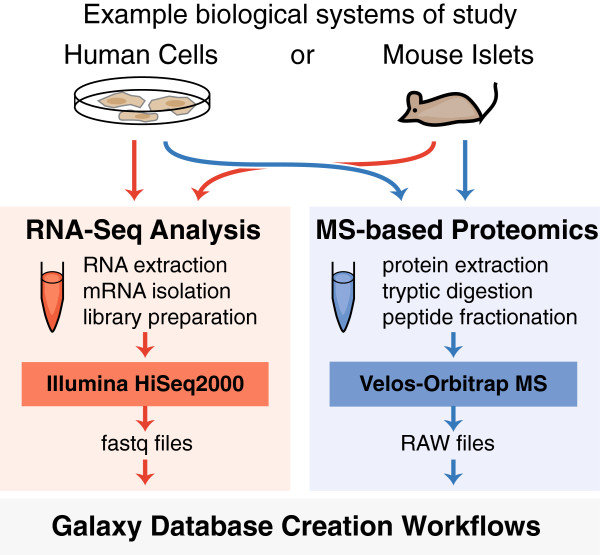
**Experimental overview.** The Galaxy-P workflows take as input sample-specific RNA-Seq data and create sample-specific protein databases. These protein databases are then employed for MS-based proteomics database searching. The workflows were developed on datasets generated from human (Jurkat cells) and mouse (B6 and CAST islets) samples.

**Figure 2:**
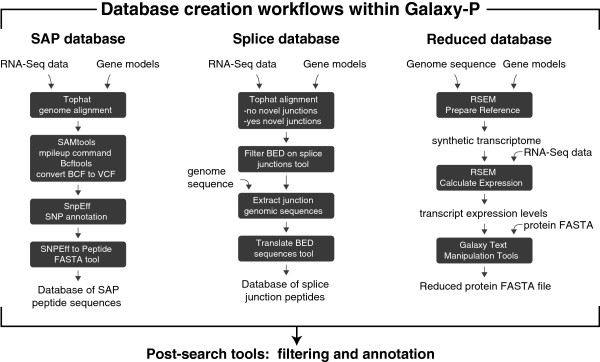
**Overview of workflows implemented in Galaxy-P that utilize RNA-Seq data for improved proteomics.** The single amino acid polymorphism (SAP) database workflow detects non-synonymous SNPs that yield SAPs. The splice database workflow detects alternatively spliced transcripts and the corresponding novel splice junction polypeptide sequences. The reduced database workflow quantifies the sample’s transcriptome, optionally removes likely unexpressed protein sequences, and allows determination of RNA-protein correlations. Post-search tools filter and annotate novel peptides.

We demonstrated the utility of these workflows on parallel RNA-Seq and proteomics datasets collected from the same sample. Figure 1 shows an overview of the experimental design employed to collect RNA-Seq and proteomic data from human Jurkat cells and mouse pancreatic islets from B6 and CAST mice. From each sample, paired-end RNA-Seq reads (350 bp, 2 × 100bp) from polyadenylated mRNAs were sequenced on an Illumina HiSeq2000 and tandem mass spectra of tryptically digested peptides were collected on a Velos-Orbitrap mass spectrometer. Figure 2 gives an overview of the three bioinformatic workflows, which are described below. These workflows should be considered merely the starting point for more complex bioinformatic pipelines and were designed to be readily edited, extended, and evolved.

### Galaxy workflows for RNA-Seq-derived database creation

#### SAP database

SNPs are single nucleotide differences between genomes of different individuals and are one of the most common types of genetic variation [18]. SNPs that reside within a protein coding region and change the coding amino acid are termed non-synonymous SNPs (nsSNPs) and the corresponding amino acid is then called a single amino acid polymorphism (SAP). Since a change in protein coding sequence can potentially alter a protein’s function, it is important to directly measure SAP-containing proteins by mass spectrometry. This would allow the evaluation of the post-translational consequences of a given variant. For example, the quantification of each SAP-containing peptide derived from a heterozygous gene pair could allow for measurement of allele-specific protein expression. These values may be compared with allele-specific RNA abundance values to study potential translational regulation of specific alleles [19].

Most reference protein databases contain only those amino acid sequences that are translated from the reference genome, which typically represent nucleotide sequences derived from one or more representative individuals or strains [20]. Therefore, SAPs present in a particular experimental sample will be missed unless they are explicitly added to the database. To solve this problem, we and others have shown that customized SAP polypeptide databases can be constructed from RNA-Seq data. The set of nsSNPs encoded in a sample’s transcriptome can be detected by RNA-Seq and the stretches of RNA sequences containing nsSNPs can be translated into SAP-containing protein sequences for database searching [4, 10].

The SAP database workflow in Galaxy-P inputs raw RNA-Seq data and outputs a database of SAP polypeptide entries that can be used for MS searching. The workflow aligns RNA-Seq reads to the reference genome using Tophat [21], calls SNPs using SAMtools [22], and annotates the SNPs that reside within protein-coding regions using SNPeff [23]. To convert the annotated SNPs into a SAP-containing polypeptide database, the workflow uses a tool we developed within Galaxy-P called “SNPeff to Peptide Fasta”. Within this tool, the user specifies the number of amino acids to the left and right of each detected SAP to include in the final SAP database. Each entry in the database contains an informative header specifying the location of both the SNP and SAP on the transcript and protein, respectively. Additionally, if the user would like to employ an alternative SNP calling tool, like GATK, they can modify the workflow to include it [24].

We used the Galaxy-P SAP database workflow to create and employ custom SAP databases for the human and mouse samples. Using the human RNA-Seq dataset, this workflow produced a SAP database comprising 6,168 SAP polypeptide entries, which was combined with the Ensembl reference proteome. After MS database searching, 522 SAP peptides that mapped up to 491 unique SNP sites on the genome were identified. These SAP peptides would not have been detected if only the canonical Ensembl protein sequences were used for database searching. When comparing the SAP peptides detected in the present study (522) with SAP peptides detected using our previously published SAP workflow (491) [4], which used different gene models (RefSeq instead of Ensembl), there was an 89% overlap in peptide identifications.

**Figure 3:**
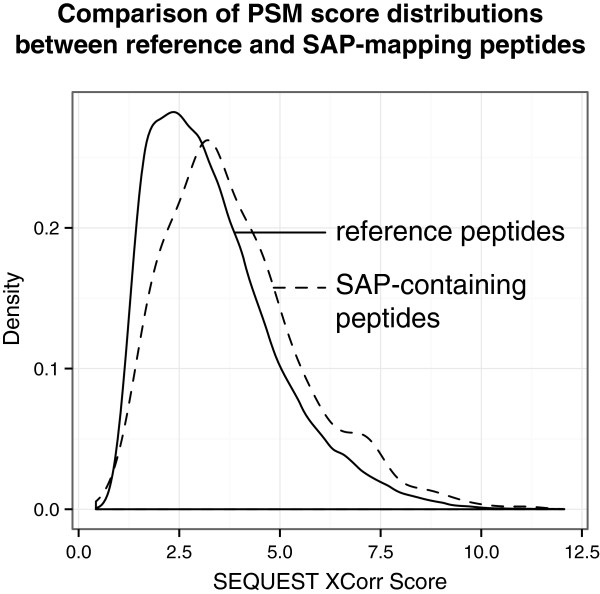
**Comparison of score distributions of all peptides identified in the search versus peptides containing SAPs.** For Jurkat cells, the distribution of SEQUEST XCorr Scores for peptides passing a 1% false discovery rate were compared between 1) peptides mapping to the Ensembl reference proteome, and 2) peptides containing single amino acid polymorphisms (SAPs) derived from the sample-matched RNA-Seq data. SAP-containing peptides had, on average, higher peptide spectral match (PSM) quality scores as compared to those of reference peptides, attesting to the high quality of the sample-specific SAP database employed for MS searching.

The peptide spectral match scores for SAP peptides were higher on average than for peptides that mapped to the reference proteome, underscoring the high quality (i.e. fewer spurious SAPs) of SAP databases derived from RNA-Seq data (Figure 3). These results are in direct contrast to previously published studies in which the SAP database was derived from the full collection of non-synonymous SNPs from repositories such as dbSNP and COSMIC. When these aggregate databases were employed for MS searching, the resultant SAP peptide identifications tended to have low scores as compared to reference peptide identifications, because a high number of SAP sequences were included in the database but not present in the analyzed samples [25].

We also demonstrated the utility of this SAP database workflow on two mouse strains, B6 and CAST. For B6, the workflow produced, as expected, only 1 SAP entry, a likely false positive or recent mutation since the mouse reference genome is based on B6 [26]. For CAST, however, the workflow output a database with 476 SAPs, which was concatenated with the Ensembl reference proteome and subsequently used for MS searching. 22 SAP peptides mapping to 19 unique SNP sites were identified. The difference between B6 and CAST SAP databases illustrates that the number of SAPs detected is dependent on the relationship between the sample and the reference genome. B6, which is in fact the strain from which the reference genome is based, did not have detected variants while CAST, a less well characterized disease model system for Type II diabetes, had many. This illustrates the importance of utilizing RNA-Seq data for proteomics analysis, especially for organisms, strains, and disease models that have not been thoroughly characterized or contain sparsely annotated reference proteomes.

Results for both human and mouse data are summarized in Table 1.

**Table 1:** **Results from creating SAP databases and using them for searching proteomic datasets**

Sample	SAP database		Proteomic identifications	
	SAPs	SNP sites	SAP Peptide IDs*	SNPs ID’d
Jurkat human cells	9,168	6,924	522	491
B6 mouse islets	1	1	N/A	N/A
CAST mouse islets	476	249	22	19

*peptide passing a 1% FDR.

#### Splice database

A majority of genes in higher eukaryotes are alternatively spliced resulting in the production of multiple mRNA forms from the same gene. The spliceosome processes pre-mRNAs by excising introns and combining specific exons to produce a mature RNA. The ubiquity of splicing, especially in humans, has been revealed by next generation sequencing methods that allow unbiased, global characterization of splicing in many cell and tissue types [27, 28].

Despite the high number of novel splice forms detected at the transcript level, proteomic databases for MS searching are far from complete in terms of splicing. There are still novel splice events in certain cell types or disease models that are not yet annotated. Consequently, the polypeptide sequences corresponding to these novel splice sites are not in the protein reference database and are thus missed during standard MS-based proteomic analyses.

Within Galaxy-P, we have created a workflow for the detection and subsequent incorporation of novel splice sequences into custom splice-junction databases. The splice database workflow first aligns RNA-Seq data to the genome twice, first to only those splice junctions found in the Ensembl gene models and second to both the Ensembl gene models and reference genome. The output BED files, which contain the coordinates of all detected junctions, are compared to each other and only those coordinates corresponding to splice junctions not present in the gene models are retrieved. Next, the genomic sequences for each splice junction are retrieved. We developed a program within Galaxy-P, “Translate BED sequences”, which translates the splice junctions and compiles all splice-junction polypeptide sequences into a database. The user may choose to filter out splice junction entries that contain stop codons, are less than a certain length, or are below a certain expression level as measured by the RNA-Seq read depth at each splice junction.

We used the splice database workflow to create and employ custom splice-junction databases for the human and mouse samples. Using the human RNA-Seq dataset, this workflow produced a splice-junction database comprising approximately 33,000 splice-junction polypeptide entries. Previously, we have found it was important to use a stringent score cut-off for peptide spectral matches corresponding to splice junction peptides [5]. Therefore, we required the same 1% *local* FDR for splice-junction peptide identifications in the present study. After MS searching against the splice-junction database, 67 novel splice junction peptides, defined as those peptides not present in the Ensembl reference proteome, were identified. There was a 57% overlap of splice-junction peptides identified in this and a previous study, which used a similar though not identical workflow (e.g. RefSeq gene models) [5].

Application of the workflow for analysis of the mouse islet RNA-Seq data resulted in a splice junction database containing approximately 32,000 (B6) and 20,000 (CAST) splice junction polypeptides. After MS searching, 58 (B6) and 72 (CAST) novel splice junction peptides were identified at a 1% local FDR.

Results for human and mouse data are summarized in Table 2. These results show that many sample-specific peptides derived from novel alternative splice events are missed when using only the reference protein database for MS searching.

**Table 2:** **Results from creating splice junction databases and using them for searching proteomic datasets**

Sample	Splice database		
	Size	Min. depth	Peptide IDs*
Jurkat human cells	33,372	6	67
B6 mouse islets	57,587	4	64
CAST mouse islets	43,244	4	66

*peptide passing a 1% local FDR.

#### Reduced database

Target decoy search strategies are widely used in mass spectrometry-based proteomics to determine a false discovery rate (FDR) for peptide identifications [29]. The underlying assumption in this approach is that the target database, which comprises the sequences of the proteins in a reference database, reflects the protein sequences actually present in the sample. However, this is rarely the case; for example, human cells have been found to express fewer than 50% of the proteins encoded in their genome at any given time [30–32]. RNA-Seq data can be employed to quantify transcripts and then remove those protein sequences from the reference database that have minimal or undetected mRNA expression levels [33]. This procedure can be thought of as reduction of database “noise” resulting from removal of putatively unexpressed proteins. This produces a smaller, sample-specific “reduced” database that could improve the number and quality of peptide identifications or could aid in disambiguation of proteins during protein inference [10, 12, 13].

In the reduced database Galaxy-P workflow, the sample-matched raw RNA-Seq data serves as input and RSEM [34] is used to quantify transcripts based on Ensembl gene models (e.g. GTF file). The output is a list of each transcripts’ abundance in Transcripts Per Million (TPM). Next, Galaxy Text Manipulation tools are used to link each protein entry in the protein FASTA file to its corresponding transcript and the transcript’s abundance in TPM.

We used the human and mouse datasets to test the reduced database workflow by creating reduced databases comprised of only those proteins with transcript abundances above 1 TPM. For human, the Ensembl protein database was reduced from approximately 104,000 to 83,000 entries. The MS search against this reduced database yielded 313 more peptide identifications as compared to the original database search. For mouse, the Ensembl protein database was reduced from approximately 52,000 to 18,000 (B6) or 17,000 (CAST) entries, increasing the number of peptide identifications for each strain by 166 (B6) and 146 (CAST). Full results for the reduced databases are listed in Table 3. Though these increases in peptide identifications are modest, this workflow offers a starting point for investigators interested in studying the relationship between database size and proteomics search results or how incorporation of transcriptional abundance values in peptide or protein identification scoring could improve database searching. One can easily change the TPM cut-off employed for various proteomic datasets that have different depths of coverage; this could allow exploration of where the optimum “balance” between including and excluding protein sequences should be. If available, alternative gene models besides Ensembl can be used, as can different transcript quantification programs.

**Table 3:** **Results from MS searching with the original Ensembl protein database and the reduced database**

Sample	RNA-Seq reads	Mass spectra	Original database		Reduced database		
			# entries	Peptide IDs*	# entries	Peptide IDs*	% increase
Jurkat human cells	80 M	500 K	104,310	73,123	82,101	73,436	0.4
B6 mouse islets	94 M	250 K	52,165	30,212	18,052	30,220	0.3
CAST mouse islets	126 M	250 K	52,165	28,902	16,940	28,823	0.2

*peptide passing a 1% FDR.

Additionally, this workflow can be used to measure RNA-protein expression correlations, because the transcriptional abundance is reported for each protein. One could perform an MS database search against the full (or reduced) protein database and then compare estimated protein abundances (e.g. from spectral counting) to the abundance of the corresponding transcript. Comparison of transcript and protein abundance levels would pinpoint proteins that are high in cellular abundance but low in transcript abundance and vice versa. This information could help researchers gain biological insight by revealing underlying mechanisms of post-transcriptional regulation of protein expression and/or turnover [35].

## Conclusions

Using RNA-Seq data to enhance MS analysis is a promising strategy to discover novel peptides specific to a sample and, more generally, to improve proteomics results. The main bottleneck for widespread adoption of this strategy has been the lack of easily used and modifiable computational tools. We provide a solution to this problem by introducing a set of workflows within Galaxy-P that easily convert raw RNA-Seq data into proteomic databases. Development within Galaxy-P brings unique benefits due to the inherent characteristics of the Galaxy-framework [15–17], such as easy publication and sharing of complete workflows with other users. Flexibility is a key benefit, as users can easily customize workflows to account for sample- or experiment-specific parameters, and also incorporate emerging new tools as desired. Although the complete workflows are available for use on the public Galaxy-P instance (i.e. implementation), the tools used and developed here are either already a part of the main Galaxy build or have been deposited in the Galaxy Tool Shed (http://toolshed.g2.bx.psu.edu/) under the “Proteomics” link. Thus these workflows should be usable on local Galaxy instances as well.

These workflows were tested on RNA and protein datasets that were collected in parallel from human and mouse samples. The results show that incorporating RNA-Seq data into proteomic analyses enables discovery of novel peptides arising from genetic variation and alternative splice forms, improves the number and quality of peptide identifications, and enables measurement of RNA-Protein expression correlations. These workflows and the benefits of the Galaxy framework provide a sound basis upon which to build newer and more sophisticated methods of RNA-Seq analysis for the continued advancement of proteomics, as newer tools and technologies arise.

## Methods

### Jurkat cell RNA-Seq

Jurkat cells were grown in 90% RPMI and 10% FBS (ATCC, Manassas, VA) to 1.3 × 10^6^ cells/mL. Total RNA was extracted using TriZol and its protocol (Invitrogen). RNA libraries were prepared using the Illumina TruSeq protocol, which includes a dT bead enrichment of polyadenylated mRNAs and size selection of 350 bp cDNA fragments. ~80 million paired end reads (350 bp, 2 × 100bp) were sequenced on an Illumina HiSeq2000. More information about this dataset may be found in [5].

### Jurkat cell MS-based proteomics

MS-based proteomics data collection has been previously described [5]. Briefly, protein was extracted and digested using the FASP protocol [36]. Peptides were fractionated on a high-pH HPLC and 28 fractions were analyzed on a nanoflow HPLC integrated with a Velos-Orbitrap mass spectrometer. The MS raw files for the Jurkat cell lysate samples are available via FTP from the PeptideAtlas data repository [37] by accessing the following link: http://www.peptideatlas.org/PASS/PASS00215.

### B6 and Cast Mouse Islet RNA-Seq

Pancreatic islets were isolated from two B6 mice and two CAST mice. Total RNA was extracted from ~250 islets of each mouse strain using the Qiagen RNeasy Mini Kit (Qiagen, Hilden, Germany). RNA-Seq data was collected as described for the human sample.

### B6 and Cast Mouse Islet proteomics

Protein was extracted from ~400 B6 islets (~470 CAST islets), and then proteomics data was collected in the same manner as for the human sample, except that 9 fractions were collected during the high-pH HPLC fractionation. MS raw files for the mouse samples are available via FTP from the PeptideAtlas data repository [37] by accessing the following link http://www.peptideatlas.org/PASS/PASS00470.

### Workflows

Three workflows were created within Galaxy-P that allow for the conversion of RNA-Seq data into customized protein databases. Galaxy-generated visualizations of these workflows may be found in Additional files 1, 2, 3, 4, 5, 6, 7. Full computational details of these workflows can be found in the following links:

#### SAP database workflows

Human_SAP_DB_Workflow.html (Additional file 1).

Mouse_SAP_DB_Workflow.html (Additional file 2).

URL to workflow within Galaxy Toolshed: http://toolshed.g2.bx.psu.edu/view/galaxyp/proteomics_rnaseq_sap_db_workflow.

#### Splice database workflows

Human_Splice_DB_Workflow.html (Additional file 3).

Mouse_Splice_DB_Workflow.html (Additional file 4).

URL to workflow within the Galaxy Toolshed: http://toolshed.g2.bx.psu.edu/view/galaxyp/proteomics_rnaseq_splice_db_workflow.

#### Reduced database workflows

Human_Reduced_DB_Workflow.html (Additional file 5).

Mouse_Reduced_DB_Workflow.html (Additional file 6).

URL to workflow within the Galaxy Toolshed: http://toolshed.g2.bx.psu.edu/view/galaxyp/proteomics_rnaseq_reduced_db_workflow.

### Database searching of mass spectrometry data

For each of the three sample types described above (human, mouse B6, mouse CAST), Galaxy-P workflows generated a SAP, splice, and reduced database which was concatenated with the cRAP database of common MS contaminants (ftp://ftp.thegpm.org/fasta/cRAP). The resultant reduced + SAP + splice + cRAP databases, one created for each of the three samples, were searched against the sample-matched raw mass spectra data using the Percolator search node within Proteome Discoverer (v1.4, Thermo Fisher Scientific, San Jose, CA). Default peaklist-generating parameters were used. Precursor m/z tolerance was set to 10 ppm and product m/z tolerance was set to 0.05 Da. Peptides with up to two missed cleavages (trypsin) were permitted. Variable methionine oxidation and static carbamidomethylation were used. Using reversed sequences as a decoy database, peptides passing a 1% global FDR were accepted as identified (except in cases where a more stringent 1% *local* FDR was mentioned in the text).

### Post-search peptide filtering and annotation

Peptide identifications were filtered using the “Filter In Reference” tool we developed within Galaxy-P, which finds and annotates the novel peptides not listed in the reference proteome. An example workflow may be found in the following links:

Example_Novel_Peptide_Filter.html (Additional file 7).

URL to workflow within the Galaxy Toolshed: http://toolshed.g2.bx.psu.edu/view/galaxyp/proteomics_novel_peptide_filter_workflow.

## Availability of supporting data

The MS raw files for the Jurkat cell lysate samples are available via FTP from the PeptideAtlas data repository [37] by accessing the following link: http://www.peptideatlas.org/PASS/PASS00215. Information for download of the RNA-Seq data collected from Jurkat cell lysates may be found in a previous publication [5]. MS raw files for the mouse samples are available via FTP from the PeptideAtlas data repository [37] by accessing the following link http://www.peptideatlas.org/PASS/PASS00470.

### Ethics

The Biochemistry Department at the University of Wisconsin is AALAC-approved. All animal handling procedures are pre-approved by the University’s Research Animal Resource Committee (RARC), and are in strict accordance with the standards set forth by the National Institutes of Health Office of Animal Care and Use.

## Electronic supplementary material

Additional file 1: **Human SAP database workflow details.** (HTML 27 KB)

Additional file 2: **Mouse SAP database workflow details.** (HTML 24 KB)

Additional file 3: **Human splice database workflow details.** (HTML 27 KB)

Additional file 4: **Mouse splice database workflow details.** (HTML 13 KB)

Additional file 5: **Human reduced database workflow details.** (HTML 27 KB)

Additional file 6: **Mouse reduced database workflow details.** (HTML 24 KB)

Additional file 7: **Novel peptide filtering workflow details.** (HTML 27 KB)
