# Supplementary material for: Using Galaxy-P to leverage RNA-Seq for the discovery of novel protein variations
Source: BMC Genomics. 2014 Aug 22;15(1):703. doi: 10.1186/1471-2164-15-703 (PMC4158061; doi:10.1186/1471-2164-15-703)
Supplement: Supplementary file 4 — Additional file 4: Mouse splice database workflow details. (HTML 13 KB) [file 12864_2014_6401_MOESM4_ESM.html]

 Galaxy | Accessible Workflow | Example Novel Peptide Filter / Galaxy-P


Example Novel Peptide Filter Workflow

### Galaxy Workflow ' Example Novel Peptide Filter'

---

| Step | Annotation |
| --- | --- |
| Step 1: Input dataset  Ensembl pep.all (Reference Protein FASTA file) *select at runtime* |  |
| Step 2: Input dataset  cRAP database of MS contaminants *select at runtime* |  |
| Step 3: Input dataset  MS Search Peptide ID results *select at runtime* | Any MS search software may be used to generate this table-formatted (e.g. excel) list of peptide identifications as long as peptide IDs are listed under one column. |
| Step 4: Concatenate datasets  Concatenate Dataset Output dataset 'output' from step 1  **Datasets**  **Dataset 1**  Select Output dataset 'output' from step 2 | Concatenates the reference proteome (Ensembl pep.all) and the contaminants database (cRAP). |
| Step 5: FASTA-to-Tabular  Convert these sequences Output dataset 'out\_file1' from step 4  How many columns to divide title string into? 1  How many title characters to keep? 0 | Convert Reference+cRAP fasta into tabular format. Column 1: Accession Column 2: aa sequence |
| Step 6: find in reference  Input file to be filtered Output dataset 'output' from step 3  reference file to search Output dataset 'output' from step 5  select columns to compare Choose the column of input and reference to compare  column in input (defaults to last column) 2 (value not yet validated)  column in reference (defaults to last column) 2 (value not yet validated)  Ignore case when comparing True  Choose outputs lines with no match in reference  Annotate found input entries with columns from reference No | Input is the list of peptides identified from MS search software. Peptides should be in table format and listed within a column. |
| Step 7: find in reference  Input file to be filtered Output dataset 'novel' from step 6  reference file to search *select at runtime*  select columns to compare Choose the column of input and reference to compare  column in input (defaults to last column) 2 (value not yet validated)  column in reference (defaults to last column) 2 (value not yet validated)  Ignore case when comparing True  Choose outputs lines with match in reference  Annotate found input entries with columns from reference Yes  columns from reference to append to found input lines 1 (value not yet validated)  separator to place between annotations from different reference lines ;  separator to place between annotation columns from the same reference line , | Input are list of novel peptides (not present in the reference database) and this step finds to which variations each novel peptide corresponds. |
