# Supplementary material for: Using Galaxy-P to leverage RNA-Seq for the discovery of novel protein variations
Source: BMC Genomics. 2014 Aug 22;15(1):703. doi: 10.1186/1471-2164-15-703 (PMC4158061; doi:10.1186/1471-2164-15-703)
Supplement: Supplementary file 5 — Additional file 5: Human reduced database workflow details. (HTML 27 KB) [file 12864_2014_6401_MOESM5_ESM.html]

 Galaxy | Accessible Workflow | Mouse SAP DB / UMN


Mouse SAP DB Workflow

### Galaxy Workflow 'Mouse SAP DB'

Annotation: The SAP Database Workflow creates a database of polypeptide sequences containing single amino acid polymorphisms (SAPs) detected in RNA-Seq data.

---

| Step | Annotation |
| --- | --- |
| Step 1: Input dataset  RNA-Seq left mate pair fastq *select at runtime* | RNAseq Left mate pair fastq (These should be in fastqsanger format. If not, convert with "Fastq Groomer" tool.) |
| Step 2: Input dataset  RNA-Seq right mate pair fastq *select at runtime* | RNAseq Right mate pair fastq (These should be in fastqsanger format. If not, convert with "Fastq Groomer" tool.) |
| Step 3: Input dataset  Ensembl GTF file (gene models) *select at runtime* | Mus\_musculus.GRCm38.74.gtf |
| Step 4: Input dataset  The Ensembl protein FASTA file (reference proteome) *select at runtime* | Mus\_musculus.GRCm38.74.pep.all.fa |
| Step 5: Select first  Select first 100000  from Output dataset 'output' from step 1 | Limit the sequence count for demonstration and testing purposes (100000) |
| Step 6: Select first  Select first 100000  from Output dataset 'output' from step 2 | Limit the sequence count for demonstration and testing purposes (100000) |
| Step 7: Convert GFF to BED  Choose GFF file Output dataset 'output' from step 3 | Convert GTF file (in GFF or GTF format) to BED format for use in Mpileup. Only SNPs residing in protein coding regions are of interest. |
| Step 8: Tophat for Illumina  RNA-Seq FASTQ file Output dataset 'out\_file1' from step 5  Use a built in reference genome or own from your history Use a built-in genome  Select a reference genome GRCm38\_canon  Is this library mate-paired? Paired-end  RNA-Seq FASTQ file Output dataset 'out\_file1' from step 6  Mean Inner Distance between Mate Pairs 150  TopHat settings to use Full parameter list  Library Type FR Unstranded  Std. Dev for Distance between Mate Pairs 20  Anchor length (at least 3) 5  Maximum number of mismatches that can appear in the anchor region of spliced alignment 0  The minimum intron length 70  The maximum intron length 500000  Allow indel search Yes  Max insertion length. 3  Max deletion length. 3  Maximum number of alignments to be allowed 20  Minimum intron length that may be found during split-segment (default) search 50  Maximum intron length that may be found during split-segment (default) search 500000  Number of mismatches allowed in the initial read mapping 2  Number of mismatches allowed in each segment alignment for reads mapped independently 2  Minimum length of read segments 25  Use Own Junctions Yes  Use Gene Annotation Model Yes  Gene Model Annotations Output dataset 'output' from step 3  Use Raw Junctions No  Only look for supplied junctions Yes  Use Closure Search No  Use Coverage Search No  Use Microexon Search No | GTF-guided alignment of reads using Tophat. |
| Step 9: MPileup  Choose the source for the reference list Locally cached  **BAM files**  **BAM file 1**  BAM file Output dataset 'accepted\_hits' from step 8  Using reference genome GRCm38\_canon  Genotype Likelihood Computation Perform genotype likelihood computation  Phred-scaled gap extension sequencing error probability 20  Coefficient for modeling homopolymer errors. 100  Perform INDEL calling Do not perform INDEL calling  Phred-scaled gap open sequencing error probability 40  **Platform for INDEL candidates**  Set advanced options Advanced  Do not skip anomalous read pairs in variant calling False  Disable probabilistic realignment for the computation of base alignment quality (BAQ) False  Coefficient for downgrading mapping quality for reads containing excessive mismatches 0  Max reads per BAM 250  Extended BAQ computation False  List of regions or sites on which to operate Output dataset 'output1' from step 7  Minimum mapping quality for an alignment to be used 0  Minimum base quality for a base to be considered 13  Only generate pileup in region  Output per-sample read depth True  Output per-sample Phred-scaled strand bias P-value False | SAMtool's MPileup command to call SNPs within protein coding regions. |
| Step 10: bcftools view  Choose a bcf file to view Output dataset 'output\_mpileup' from step 9  Retain all possible alternate alleles at variant sites Yes  Output in the BCF format. The default is VCF. No  Sequence dictionary (list of chromosome names) for VCF->BCF conversion. No  Indicate PL is generated by r921 or before (ordering is different). No  Suppress all individual genotype information. No  Skip sites where the REF field is not A/C/G/T No  The input is VCF instead of BCF. No  Uncompressed BCF output. No  Call variants using Bayesian inference. Automatically performs max-likelihood inference only Yes  Perform max-likelihood inference only, including estimating the site allele frequency, testing Hardy-Weinberg equilibrium and testing associations with LRT. No  Call per-sample genotypes at variant sites No  Use alternate INDEL-to-SNP mutation rate, default 0.15. No  variant\_filter No  Specify scaled mutation rate for variant calling, default is 0.001. No  Output variant sites only. Yes | Convert binary call format (BCF) to variant call format (VCF). |
| Step 11: SnpEff  Sequence changes (SNPs, MNPs, InDels) Output dataset 'output' from step 10  Input format VCF  Output format VCF (only if input is VCF)  Genome source Named on demand  Snpff Version Name GRCm38.73  Upstream / Downstream length No upstream / downstream intervals (0 bases)  Set size for splice sites (donor and acceptor) in bases 1 base  Filter homozygous / heterozygous changes No filter (analyze everything)  Filter sequence changes Only SNPs (single nucleotide polymorphisms)  Annotation options  Use custom interval file for annotation *select at runtime*  Only use the transcripts in this file. *select at runtime*  Filter output Do not show DOWNSTREAM changes Do not show INTERGENIC changes Do not show INTRON changes Do not show UPSTREAM changes Do not show 5\_PRIME\_UTR or 3\_PRIME\_UTR changes  Chromosomal position Use default (based on input type)  Text to prepend to chromosome name  Produce Summary Stats True  Do not report usage statistics to server True | SnpEff to annotate detected SNPs that correspond to protein-level amino acid polymorphisms (SAPs). ( Set genome version: GRCh37.73 ) |
| Step 12: SnpEff to Peptide fasta  SnpEff generated VCF file with NON\_SYNONYMOUS\_CODING annotations Output dataset 'snpeff\_output' from step 11  Ensembl all\_pep.fa Output dataset 'output' from step 4  Preceeding AAs 66  Following AAs 66 | Translate nucleotide sequences with SNPs into peptide sequences with the Single Amino Acid Polymorphisms (SAPs). Compile into a customized SAP database compatible with MS database searching strategies. |
