# Supplementary material for: Using Galaxy-P to leverage RNA-Seq for the discovery of novel protein variations
Source: BMC Genomics. 2014 Aug 22;15(1):703. doi: 10.1186/1471-2164-15-703 (PMC4158061; doi:10.1186/1471-2164-15-703)
Supplement: Supplementary file 6 — Additional file 6: Mouse reduced database workflow details. (HTML 24 KB) [file 12864_2014_6401_MOESM6_ESM.html]

 Galaxy | Accessible Workflow | Mouse Splice DB / UMN


Mouse Splice DB Workflow

### Galaxy Workflow 'Mouse Splice DB'

Annotation: Create a FASTA file of splice-junction peptides that are inferred from RNA-Seq
data

---

| Step | Annotation |
| --- | --- |
| Step 1: Input dataset  RNA-Seq left mate pair fastq *select at runtime* | RNA-Seq left mate pair fastq (These should be in fastqsanger format. If not, convert with "Fastq Groomer" tool.) |
| Step 2: Input dataset  RNA-Seq right mate pair fastq *select at runtime* | RNA-Seq right mate pair fastq (These should be in fastqsanger format. If not, convert with "Fastq Groomer" tool.) |
| Step 3: Input dataset  GTF file (gene models) *select at runtime* | Mus\_musculus.GRCm38.74.gtf |
| Step 4: Input dataset  Reference genome FASTA file *select at runtime* | GRCm38\_canon.fa Ensembl GRCm38 reference genome fasta Only canonical chromosomes included, typically. |
| Step 5: Select first  Select first 100000  from Output dataset 'output' from step 1 | Limit the sequence count for demonstration and testing purposes (100000) |
| Step 6: Select first  Select first 100000  from Output dataset 'output' from step 2 | Limit the sequence count for demonstration and testing purposes (100000) |
| Step 7: Tophat for Illumina  RNA-Seq FASTQ file Output dataset 'out\_file1' from step 5  Use a built in reference genome or own from your history Use a genome from history  Select the reference genome Output dataset 'output' from step 4  Is this library mate-paired? Paired-end  RNA-Seq FASTQ file Output dataset 'out\_file1' from step 6  Mean Inner Distance between Mate Pairs 150  TopHat settings to use Full parameter list  Library Type FR Unstranded  Std. Dev for Distance between Mate Pairs 20  Anchor length (at least 3) 5  Maximum number of mismatches that can appear in the anchor region of spliced alignment 0  The minimum intron length 70  The maximum intron length 500000  Allow indel search Yes  Max insertion length. 3  Max deletion length. 3  Maximum number of alignments to be allowed 20  Minimum intron length that may be found during split-segment (default) search 50  Maximum intron length that may be found during split-segment (default) search 500000  Number of mismatches allowed in the initial read mapping 2  Number of mismatches allowed in each segment alignment for reads mapped independently 2  Minimum length of read segments 25  Use Own Junctions Yes  Use Gene Annotation Model Yes  Gene Model Annotations Output dataset 'output' from step 3  Use Raw Junctions No  Only look for supplied junctions No  Use Closure Search No  Use Coverage Search No  Use Microexon Search No | GTF-guided Tophat alignment allowing for detection of splice junctions not in the GTF file. |
| Step 8: Tophat for Illumina  RNA-Seq FASTQ file Output dataset 'out\_file1' from step 5  Use a built in reference genome or own from your history Use a genome from history  Select the reference genome Output dataset 'output' from step 4  Is this library mate-paired? Paired-end  RNA-Seq FASTQ file Output dataset 'out\_file1' from step 6  Mean Inner Distance between Mate Pairs 150  TopHat settings to use Full parameter list  Library Type FR Unstranded  Std. Dev for Distance between Mate Pairs 20  Anchor length (at least 3) 5  Maximum number of mismatches that can appear in the anchor region of spliced alignment 0  The minimum intron length 70  The maximum intron length 500000  Allow indel search Yes  Max insertion length. 3  Max deletion length. 3  Maximum number of alignments to be allowed 20  Minimum intron length that may be found during split-segment (default) search 50  Maximum intron length that may be found during split-segment (default) search 500000  Number of mismatches allowed in the initial read mapping 2  Number of mismatches allowed in each segment alignment for reads mapped independently 2  Minimum length of read segments 25  Use Own Junctions Yes  Use Gene Annotation Model Yes  Gene Model Annotations Output dataset 'output' from step 3  Use Raw Junctions No  Only look for supplied junctions Yes  Use Closure Search No  Use Coverage Search No  Use Microexon Search No | GTF-guided Tophat alignment with no detection of splice junctions absent from the GTF file. |
| Step 9: Filter BED on splice junctions  BED file Output dataset 'junctions' from step 7  reference bed file Output dataset 'junctions' from step 8  Extend the start position 66  Extend the end position 66 | Filter out known splice junctions, only keep the novel ones. |
| Step 10: Extract Genomic DNA  Fetch sequences for intervals in Output dataset 'novel\_junctions' from step 9  Interpret features when possible No  Source for Genomic Data History  Using reference file Output dataset 'output' from step 4  Output data type Interval | Retrieve the DNA sequences for the novel splice junctions. |
| Step 11: Translate BED Sequences  BED file with added sequence column Output dataset 'out\_file1' from step 10  Genome reference name  The SEQTYPE:STATUS to include in the fasta ID lines pep:splice  Add the bed score field fasta ID line with this tag name depth  Filter out translations with stop codons before the splice site Yes  Stop codon filtering start position base pairs 66  Stop codon filtering end position base pairs 66  Trim translations to stop codons Yes  Minimum length of a translation to be reported 10 | Output splice-junction peptide translations from DNA sequences. |
