# Supplementary material for: Using Galaxy-P to leverage RNA-Seq for the discovery of novel protein variations
Source: BMC Genomics. 2014 Aug 22;15(1):703. doi: 10.1186/1471-2164-15-703 (PMC4158061; doi:10.1186/1471-2164-15-703)
Supplement: Supplementary file 7 — Additional file 7: Novel peptide filtering workflow details. (HTML 27 KB) [file 12864_2014_6401_MOESM7_ESM.html]

 Galaxy | Accessible Workflow | Mouse Reduced DB / UMN


Mouse Reduced DB Workflow

### Galaxy Workflow 'Mouse Reduced DB'

Annotation: Filter out known peptides that don't appear in this RNAseq sample

---

| Step | Annotation |
| --- | --- |
| Step 1: Input dataset  Ensembl Genome Reference Fasta *select at runtime* | GRCm38\_canon.fa (Ensembl reference fasta with only chromosome assigned sequences) |
| Step 2: Input dataset  Ensembl GTF (gene models) *select at runtime* | Mus\_musculus.GRCm38.74.gtf |
| Step 3: Input dataset  Ensembl Protein FASTA (reference proteome) *select at runtime* | Mus\_musculus.GRCm38.74.pep.all.fa |
| Step 4: Input dataset  RNA-Seq right paired-end fastq *select at runtime* | RNA-Seq left mate pair fastq (These should be in fastqsanger format. If not, convert with "Fastq Groomer" tool.) |
| Step 5: Input dataset  RNA-Seq right paired-end fastq *select at runtime* | RNA-Seq right mate pair fastq (These should be in fastqsanger format. If not, convert with "Fastq Groomer" tool.) |
| Step 6: RSEM prepare reference  Reference transcript source reference genome and gtf  reference fasta file Output dataset 'output' from step 1  gtf Output dataset 'output' from step 2  Map of gene ids to transcript (isoform) ids *select at runtime*  reference name primaryEnsemblGtfRef  PolyA  Add poly(A) tails to all transcripts  The length of the poly(A) tails to be added. (Default: 125) 125  Disable the conversion of 'N' characters to 'G' characters in the reference sequences False | Given a GTF file and the reference genome, this tool constructs a synthetic transcriptome that will be used for isoform quantification during "-calculate expression". |
| Step 7: FASTA-to-Tabular  Convert these sequences Output dataset 'output' from step 3  How many columns to divide title string into? 1  How many title characters to keep? 0 |  |
| Step 8: RSEM calculate expression  Sample name rsem\_sample  RSEM Reference Source From your history  RSEM reference Output dataset 'reference\_file' from step 6  RSEM Input file type FASTQ  FASTQ type phred33 qualities (default for sanger)  Library type Paired End Reads  Read 1 fastq file Output dataset 'output' from step 4  Read 2 fastq file Output dataset 'output' from step 5  bowtie settings use bowtie defaults  Seed length used by the read aligner 25  Is the library strand specific? No  Additional RSEM options Use RSEM Defaults  Create bam results files No BAM results files | Given then RNA-Seq reads (fastq) and synthetic transcriptome (from "-prepare reference"), this tool quantifies the abundances of each mRNA transcript within the GTF file. |
| Step 9: Cut  Cut columns c1  Delimited by Tab  From Output dataset 'output' from step 7 |  |
| Step 10: Filter  Filter Output dataset 'isoform\_abundances' from step 8  With following condition c3>0.000001  Number of header lines to skip 0 | Selection of lower threshold of transcriptional abundance in TPM required for inclusion of the corresponding protein in the reduced database. |
| Step 11: Convert  Convert all Whitespaces  in Dataset Output dataset 'out\_file1' from step 9 |  |
| Step 12: Compute  Add expression c3\*1000000  as a new column to Output dataset 'out\_file1' from step 10  Round result? NO |  |
| Step 13: Cut  Cut columns c5  Delimited by Tab  From Output dataset 'out\_file1' from step 11 |  |
| Step 14: Convert  Convert all Colons  in Dataset Output dataset 'out\_file1' from step 13 |  |
| Step 15: Cut  Cut columns c2  Delimited by Tab  From Output dataset 'out\_file1' from step 14 |  |
| Step 16: Paste  Paste Output dataset 'output' from step 7  and Output dataset 'out\_file1' from step 15  Delimit by Tab |  |
| Step 17: Join two Datasets  Join Output dataset 'out\_file1' from step 16  using column 3 (value not yet validated)  with Output dataset 'out\_file1' from step 12  and column 1 (value not yet validated)  Keep lines of first input that do not join with second input No  Keep lines of first input that are incomplete No  Fill empty columns No |  |
| Step 18: Add column  Add this value  to Dataset Output dataset 'out\_file1' from step 17  Iterate? NO |  |
| Step 19: Add column  Add this value tpm:  to Dataset Output dataset 'out\_file1' from step 18  Iterate? NO |  |
| Step 20: Merge Columns  Select data Output dataset 'out\_file1' from step 19  Merge column 1 (value not yet validated)  with column 9 (value not yet validated)  **Columns**  **Columns 1**  Add column 10 (value not yet validated)  **Columns 2**  Add column 8 (value not yet validated) |  |
| Step 21: Cut  Cut columns c11,c2  Delimited by Tab  From Output dataset 'out\_file1' from step 20 |  |
| Step 22: Tabular-to-FASTA  Tab-delimited file Output dataset 'out\_file1' from step 21  Title column(s) 1 (value not yet validated)  Sequence column 2 (value not yet validated) | Final reduced database after application of a TPM cut-off. |
| Step 23: FASTA Width  Library to re-format Output dataset 'output' from step 22  New width for nucleotides strings 80 | Format FASTA to desired width. |
